# Recovery of Phenolic Compounds from Jackfruit Seeds Using Subcritical Water Extraction

**DOI:** 10.3390/foods12173296

**Published:** 2023-09-02

**Authors:** Ravshanbek Sultanbekovich Alibekov, Siti Mazlina Mustapa Kamal, Farah Saleena Taip, Alifdalino Sulaiman, Abdugani Mutalovich Azimov, Klara Abdyrazahovna Urazbayeva

**Affiliations:** 1 Food Engineering Department, M. Auezov South-Kazakhstan University, Tauke Khan Avenue 5, Shymkent 160012, Kazakhstan; ralibekov@hotmail.com (R.S.A.); azimov-78@mail.ru (A.M.A.); klara_abdrazak@mail.ru (K.A.U.); 2 Department of Process and Food Engineering, Universiti Putra Malaysia, Serdang 43400, Malaysia; farahsaleena@upm.edu.my (F.S.T.); alifdalino@upm.edu.my (A.S.)

**Keywords:** jackfruit seed, subcritical water extraction, phenolic compounds, antioxidant activity

## Abstract

Jackfruit is one of the major fruits cultivated in many Asian countries. Jackfruit seeds are generally disposed of into the environment, which causes an environmental concern that leads to biowaste accumulation. The seeds have excellent nutritional value, such as carbohydrates, protein, fats, minerals, and bioactive compounds. Bioactive compounds, such as phenolic, can be recovered from jackfruit seeds that could add value to the food and pharmaceutical industry. Thus, this study focused on utilizing subcritical water to extract the phenolic compounds from jackfruit seeds and correlate them with antioxidant activity (AA). The extraction of phenolic compounds was studied at different temperatures and extraction times. The highest total phenolic compounds (TPC) and AA were obtained by treating the jackfruit seed powder at 210 °C, 30 min, and 15% solid loading under subcritical water extraction (SWE) with 1.84 mg GAE/100 g (TPC) and 86% (AA). High correlation between the extracted TPC and AA of the jackfruit seed extracts was obtained (R^2^ = 0.96), indicating a significant positive relationship between TPC and AA. A higher amount of TPC was obtained via SWE as compared to Soxhlet extraction (1 h:0.53 mg GAE/100 g and 4 h:1.20 mg GAE/100 g). More pores were detected on the surface of the sample treated by SWE than using Soxhlet extraction. Thus, jackfruit seed extracts can be potentially beneficial in the fortification of fermented dairy or meat products.

## 1. Introduction

Jackfruit is among the major tropical fruits planted worldwide and comprises three parts: rind, pulp, and seeds. Jackfruit can be considered a functional food because of the abundance of valuable compounds contained in different parts of the fruit [1]. Thus, its demand has increased due to its nutritional benefits [2]; however, only 25–35% is edible, and 65–75% is discarded as waste and by-products [3]. The processing of jackfruit continuously generates biowaste that leads to increased environmental problems. To reduce the accumulation of this biowaste, the valorization of this waste or by-products to high-value products is needed. Jackfruit consists of 8–15% seeds [4,5], which is the most common waste rich in proteins, carbohydrates, minerals, starch, and vitamins [3,6]. The seeds also contain many phytochemicals that include bioactive substances, such as phenolic compounds [7], flavonoids [8], and tannins [8], that make it potent in antioxidant, anti-bacterial, anti-microbial, and anti-inflammatory uses, as well as many other applications in the food and pharmaceutical industries.

Phenolic compounds are key contributors to antioxidant capacities since they possess the capacity to donate an electron or hydrogen to produce stable radical intermediates. Antioxidants are chemical compounds that possess the ability to delay or impede the oxidation of lipids and other molecules by intervening in the initiation or progression of oxidizing chain reactions [9,10]. These antioxidants can be broadly categorized into two groups: natural and synthetic [11]. In recent times, there has been a notable surge in interest in the search for naturally occurring antioxidants that can be incorporated into food products or medicinal materials, as a replacement for synthetic antioxidants, which are restricted due to concerns over their carcinogenic potential [11]. Among the available natural sources, raw fruits and vegetables have been identified as the primary reservoirs of antioxidants for human consumption [12]. This growing focus on natural antioxidants stems from their potential health benefits and their suitability as safer alternatives to synthetic counterparts in various applications. Thus, it is interesting to correlate the phenolic compounds obtained from jackfruit seeds with antioxidant activity.

Compared to other wastes, phenolic content in waste extracts usually shows a significant increase during thermal processing; hence, it could add more value to the processed waste [13]. The extracted antioxidants and phenolics can be used to fortify many types of food that include yogurt [14], ice cream [15], edible oils [16], seafood [17], and meat products [18]. They have been proven to improve the taste of cheese at low concentrations. Phenolic compounds are also able to improve certain functional properties of milk and dairy products, in terms of the stability to oxidation and less susceptibility to microbial growth [19]. The application of these bioactive compounds is not limited to food but also to developing different types of functional food [20] and bioactive packaging [21] and even improving the physicochemical properties of frying oil [22].

To recover these bioactive compounds, many extraction processes have been utilized to obtain high yield and high-purity extracts. Common extraction techniques use time-consuming conventional methods, such as maceration, Soxhlet extraction, and solvent extraction, which utilize organic solvents (e.g., ethanol, methanol, acetone) [7,23]. Organic solvents can pose environmental risks due to their potential to contaminate water, soil, and air, and their use should be minimized or replaced with more eco-friendly alternatives for sustainable practices. Thus, this study aimed to reduce the use of organic solvents by utilizing the subcritical water extraction (SWE) technique. SWE is known to produce higher yields in more reduced processing time than traditional methods and offers an innovative way to increase the production of bioactive compounds [24,25]. The SWE process requires the application of water under high pressure to maintain the water in a liquid state at a temperature above its normal boiling point [25,26]. As the temperature increases, its dielectric constant, surface tension, and viscosity will reduce [27], but its diffusivity will improve. It was reported that, at 25 °C, the dielectric constant of water is 80, but when the temperature rises to 250 °C, the dielectric constant falls to 25, which is between methanol (e = 33) and ethanol (e = 24) [26]. Therefore, the dielectric constant of water can be varied by varying the temperature and pressure [28]. Another advantage of SWE is that the process time can range from 1 to 50 min, which is a shorter time compared to the maceration process. Thus, this technology offers an environmentally friendly and innovative way to increase the production of bioactive compounds. Controlling process parameters such as temperature, time, and pressure could facilitate the extraction process with a high yield of bioactive compounds. 

Therefore, this study focused on recovering bioactive compounds, especially phenolic content, from jackfruit seeds and correlating them with antioxidant activity. Jackfruit seeds are well-known for their high antioxidant activity and other therapeutic characteristics with potential disease prevention capability [29]. Thus, outcomes from this study will enhance the antioxidant extraction procedure from jackfruit seeds and, at the same time, facilitate the valorization of fruit waste. 

## 2. Materials and Methods

### 2.1. Raw Materials and Reagents

Jackfruits were collected from Seri Kembangan, Selangor, Malaysia. The jackfruit seeds were taken out from the flesh and peeled off their seed coats. Then, the seeds were cleaned thoroughly using water and dried at 80 °C for 8 h using an oven to reduce the moisture content to less than 10% [30]. The dried jackfruit seeds were ground using a blender and sieved with a 250 μm mesh sifter. The jackfruit seed powder was kept in a glass bottle sealed with aluminum foil and was placed in a desiccator for further analysis. 

All chemicals used in this research were analytical grade. (i) Sodium carbonate and Folin and Ciocalteu’s Phenol Reagent were obtained from R&M Chemicals, Selangor, Malaysia. (ii) DPPH (2,2-Diphenyl-1-picrylhydrazyl) (free radical), gallic acid, and methanol were obtained from Fisher Scientific, Selangor, Malaysia. (iii) Ferulic acid was purchased from Sigma Aldrich, Selangor, Malaysia. 

### 2.2. Soxhlet Extraction

Extraction was carried out using Soxhlet equipment that consisted of a 250 mL round bottom flask containing 153 mL of the extraction solvent (pure methanol). A 27 g sample was placed into a filter paper and inserted into the flask. The extractions were performed for 1 h and 4 h, and the extraction temperature was kept at the solvent boiling point (for methanol ~65 °C). Then, the extracts were centrifuged (KUBOTA Corporation, Osaka, Japan) at 4000 rpm for 10 min and filtered using syringe filter nylon (0.22 mm) to separate the solid residue and filtrate. The filtrates were then analyzed for the total phenolic compounds, antioxidant activity, and phenolic acid analysis. The solid residue was stored in a freezer with a temperature of −25 °C for surface morphology analysis. 

### 2.3. Subcritical Water Extraction

Subcritical water extractions were performed using a batch fluid extraction system that consisted of a salt bath. The salt bath, containing a ratio of 1:1 of potassium nitrate and sodium nitrite, was prepared as the heating medium for the subcritical water extraction. It was pre-heated until the temperature reached the setting range of 180–240 °C, and process time ranged from 10 to 30 min. The pressure range was from 1.00 MPa to 3.35 MPa for the respective temperature range.

All experiments were conducted with a constant solid loading of 15% (*w*/*w*). Solid loading was the mass of sample powder divided by the total mass of solution and multiplied by 100% [31]. Then, the sample and water as solvent were inserted into a 10 mL stainless-steel reactor cell. Argon gas was blown into the reactor cell for 1 min to remove all the air. After that, the reactor was immersed in a preheated salt bath (180, 210, and 240 °C) to commence the reaction after reaching the extraction time (10, 20, and 30 min). To stop the reaction, the reactor cell was taken out of the bath and quickly quenched in cold water for 20–24 h. The basis for temperature and process time range was based on the previous studies on SWE [31,32,33] with some modifications. The extracts were centrifuged (KUBOTA Corporation, Japan) at 4000 rpm for 10 min and filtered through a filter of 0.22 µm. The crude extract and solid residue were stored at −25 °C for further analysis. 

### 2.4. Determination of Total Phenolic Content

The phenolic content of the extract was determined by a modified Folin–Ciocalteu method [31,34]. The extract (0.2 mL) was prepared up to 3 mL with distilled water and mixed with Folin–Ciocalteu reagent (0.5 mL) for 3 min, followed by the addition of 2 g/100 mL (*w*/*v*) of sodium carbonate (2 mL). The extract solution was vortexed for 20 s prior to incubating for 60 min in the dark, and the absorbance of the extract solution was measured at 765 nm by UV-Vis spectrophotometry (Ultrospec 3100 Pro, Amersham Biosciences Corp., Boston, MA, USA). The phenolic content was then calculated from the gallic acid calibration curve. Gallic acid was used as a reference standard, and the results were expressed as milligram gallic acid equivalent per dry weight of jackfruit seed powder (mg GAE/g). All experiments were performed in duplications. 

### 2.5. Antioxidant Activity Analysis

The antioxidant activity of the jackfruit seed extract was measured using the 1,1-diphenyl-2-picryl-hydrazyl (DPPH) test, which was amended slightly [30]. A total of 3 mL of extract solution was added with 5 mL of 0.1 mM DPPH in methanol. After that, the solution was vortexed for 20 s and incubated at room temperature for 30 min in the dark. The control solution consisted of 0.1 Mm DPPH in methanol. The blank solution was methanol only. The absorbance was measured at a 517 nm wavelength using a UV-Vis spectrophotometer [31]. The antioxidant activity analysis of all samples was determined using the formula below:

(1) $$\mathrm{Antioxidant}\;\mathrm{activity}\;\mathrm{of}\;\mathrm{sample}\;\left(\%\right)\;=\;\frac{{\mathrm{Absorbance}}_{\mathrm{control}}-{\mathrm{Absorbance}}_{\mathrm{sample}}}{{\mathrm{Absorbance}}_{\mathrm{control}}}\;\times \;100\%$$

### 2.6. High Performance Liquid Chromatography (HPLC) Analysis

HPLC analysis was performed to determine the phenolic acid content (gallic acid, p-coumaric acid, caffeic acid, and ferulic acid) from the jackfruit seed extracts. The HPLC (Prominence) was equipped with LUNA C-18 column (5 µm, length = 250 mm, diameter = 4.6 mm) with a UV detector (SPD-20A Prominence, Shimadzu) and a flow rate of 0.5 mL/min. The mobile phase consisted of three solvents: water (A), acetonitrile (B), and acetic acid (94:6, *v*/*v*, pH 2.3). The solvent gradient was designed to be 0–15% B in 40 min, 15–45% B in 40 min, and 45–100% B in 10 min. The samples and mobile phases were filtered via a 0.22 µm filter before being injected into the HPLC. The quantities of phenolic acid in the extracts were determined by comparing their retention time and the spectral data of UV-diode array detection at 280 nm and using a series of standard solutions (gallic acid, p-coumaric acid, caffeic acid, and ferulic acid).

### 2.7. Fourier Transform Infrared Spectroscopy (FTIR)

Fourier-transform infrared spectroscopy was used to identify the potential chemical functional groups in the jackfruit seed extracts using the attenuated total reflectance (ATR) approach. The method is predicated on determining functional groups inside molecules that vibrate by stretching or bending in different ways when exposed to specific light wavelengths [35]. The extracts (samples) were put on the sample holder, and the IR spectra were obtained using an FTIR Spectrometer (Perkin Elmer) with the Spectrum 100 and with an ATR detector. The samples were scanned with a wavenumber range of 4000–650 cm^−1^ [36]. Wavenumber is 1/wavelength and correlates to the energy of molecular bond vibration. An FTIR spectrum is created by plotting the vibrations and intensity (% transmission) against the frequency of light (cm^−1^) that is applied to the material [35].

### 2.8. Surface Morphological Analysis

The surface morphologies of jackfruit seed powder before and after extraction were conducted using JSM—IT 100 InTouchScope^TM^ (Tokyo, Japan) scanning electron microscopy (SEM) operated with 10 kV of voltage. The dried residues were placed on a specimen holder using an adhesive and covered with an ultrathin coating of electrically conducted material, which was gold at 20 mA. The surface of the specimen was scanned by the electron beam to obtain the image [31]. 

### 2.9. Statistical Analysis

All the data (total phenolic content, antioxidant activity, and phenolic acids) were collected in at least two replications and analyzed using a two-way analysis of variance (ANOVA) test with *p* < 0.05 (95% level of significance). ANOVA was used to identify the significance of factors and evaluate the overall model fit. All statistical analyses were conducted using Minitab 17.1 software.

## 3. Results and Discussion

### 3.1. Effect of Process Parameters on Total Phenolic Compounds (TPC) and Antioxidant Activity (AA) of Jackfruit Seed Extract

The results of total phenolic compounds and the antioxidant activity of the jackfruit seeds extracted from the subcritical water extraction process in the temperature range of 180–240 °C and time range of 10–30 min are shown in Table 1; they were found using the one-factor-at-a-time (OFAT) method. Analysis of variance (ANOVA) tests were used to evaluate the study of the extraction of jackfruit seeds using subcritical water with the TPC and AA responses. 

**Figure 1:**
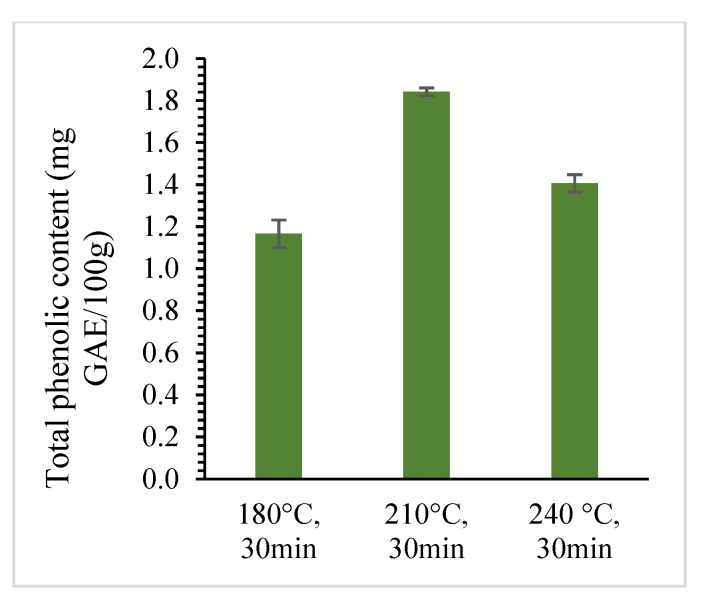
The effect of temperature on total phenolic content using SWE for 30 min and 15% solid loading.

**Figure 2:**
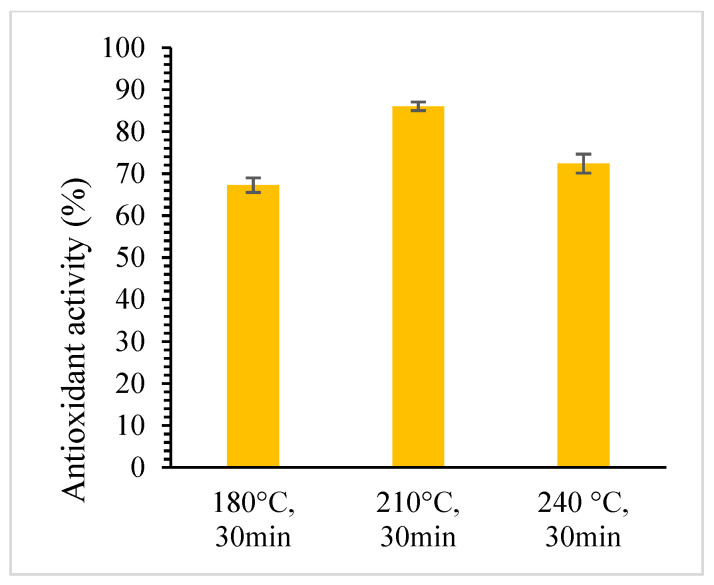
The effect of temperature on antioxidant activity using SWE for 30 min and 15% solid loading.

Jackfruit seed powder was treated under SWE at different temperatures, ranging from 180 °C to 240 °C, and times, ranging from 10 to 30 min, with a constant solid loading of 15%. Figure 1 and Figure 2 show that the total phenolic compounds and antioxidant activity of the extracts increases when temperature increased from 180 °C to 210 °C but decreased as extraction temperature increased to 240 °C. The decrease in the yield of total phenolic compounds might be because the extended exposure of the active substances to high temperatures induced breakdown and structural degradation over longer extraction durations. The highest total phenolic compound and antioxidant activity were found to be 1.84 ± 0.02 mg GAE/100 g and 86.00 ± 1.01% at 210 °C for 30 min. However, the lowest yield was found at 180 °C for 10 min with the total phenolic content of 0.84 ± 0.04 mg GAE/100 g and antioxidant activity of 61.29 ± 1.72%. At low temperatures, the movement of compounds within the solid matrix may be hindered, leading to lower extraction efficiency and yield. Similar behaviors were observed when SWE was used to extract phenolic compounds from red dates [37] and microalgae [36]. 

Water polarity and phenolic chemical solubility may both have an impact on how total phenolic chemicals are affected by temperature. Water had different dielectric constants at different temperatures during the SWE process, resulting in different polarity. A low dielectric constant indicates that the hydrogen connections between water molecules have been disrupted. Water’s hydrogen bond is broken, enhancing nonpolar solutes’ solubility [38]. Raising the temperature can potentially improve the phenolic compound solubility and diffusion coefficients that permit a faster extraction rate. As temperature increases, it also lowers the water viscosity and surface tension, whereas diffusivity is raised, permitting more solvent penetration into the matrix and improving extraction efficiency and speed [36]. However, at high temperatures (in this study at 240 °C), the extraction of undesirable impurities and the decomposition of thermolabile components [39] may occur. 

From Table 1, the extracts’ total phenolic compounds and antioxidant activity increased with the extraction time ranging from 10 to 30 min for temperatures ranging from 180 °C to 210 °C. The highest TPC and antioxidant activity were obtained by treating the jackfruit seed powder at 210 °C, 30 min, and 15% solid loading under SWE. On the other hand, the lowest total phenolic content and antioxidant activity were at 180 °C for 10 min. The extraction time influenced the duration of the jackfruit seed powder being exposed to the subcritical water. A longer extraction time allowed for better diffusion of compounds from the solid matrix into the solvent. Too short an extraction time may result in insufficient time for the water to enter the jackfruit seed powder and adequately extract the required compounds, resulting in reduced yields. Phenolic compounds are often present in intricate structures, such as cell walls, or bound to other components; therefore, extending the extraction time allows for the complete breakdown of these complex structures, resulting in a higher extraction yield of the desired compounds. However, at 240 °C, there was a decreasing trend, as extraction time increased from 10 to 30 min. This could be due to the extended exposure of the compounds to high temperatures, inducing breakdown and structural degradation over an extended extraction time. Moreover, certain polyphenols may polymerize and lessen the free polyphenols in the extracts, causing a poor polyphenol yield [40]. In contrast, Kheirkhah et al. [41] found that the best phenolic compound recovery from SWE was at 200 °C and a longer extraction time, which was 90 min for kiwifruit pomace. This might be due to different structures of the cell walls for different kinds of fruits.

### 3.2. Correlation between Total Phenolic Compounds and Antioxidant Activity of Jackfruit Seeds Extract

**Figure 3:**
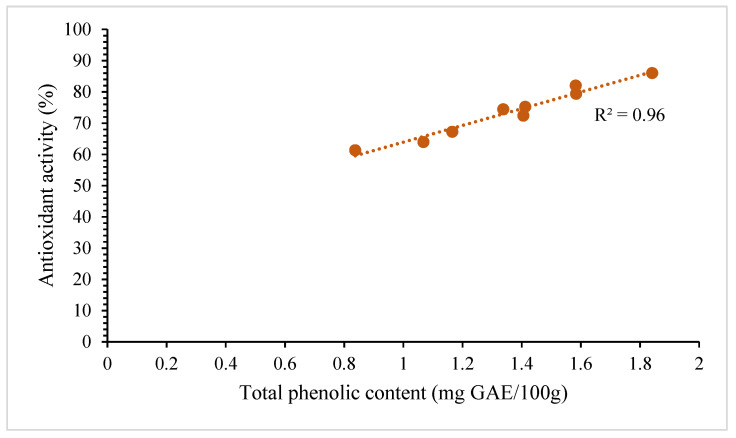
The correlation between the total phenolic content and antioxidant activity of jackfruit seed extract using SWE (R^2^ = 0.96).

All results of the total phenolic compound and antioxidant activity of each operation parameter were analyzed to evaluate the correlation between the extracted phenolic content and antioxidant activity of the jackfruit seed extracts using the data obtained from statistical analysis. Figure 3 shows an R^2^ value of 0.96 that indicates a significant positive relationship between total phenolic compounds and antioxidant activity during subcritical water extraction. Changes in total phenolic compounds explained about 96% of the variability in antioxidant activity. Higher antioxidant activity was related to increased total phenolic compounds, which is due to the ability of phenolic compounds to donate a hydrogen atom or electron to form stable radical intermediates, and they perform as major contributors to antioxidant capacities [42]. The results are supported by the studies conducted by Yamin et al. [43] on jackfruit peel and Zzaman [44] on jackfruit seeds, which also found a high correlation between total phenolic compounds and antioxidant activity. Hence, the total phenolic content can be utilized to perform quick antioxidant activity screening because of the high correlation with antioxidant activity. Jackfruit seeds exhibit significantly higher levels of phenolic compounds, likely attributed to the antioxidative properties of these phenolics, which play a crucial role in preserving the species’ overall health and development.

### 3.3. Comparison between Subcritical Water Extraction with the Soxhlet Extraction

**Figure 4:**
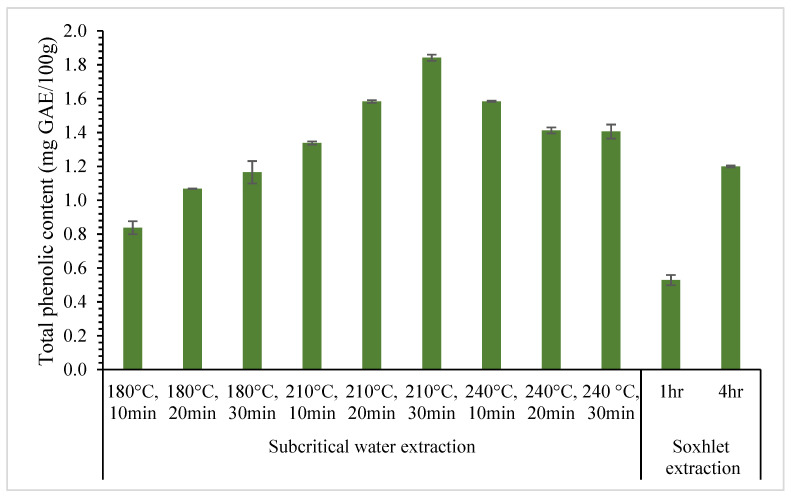
Total phenolic content comparison between SWE and Soxhlet extraction.

**Figure 5:**
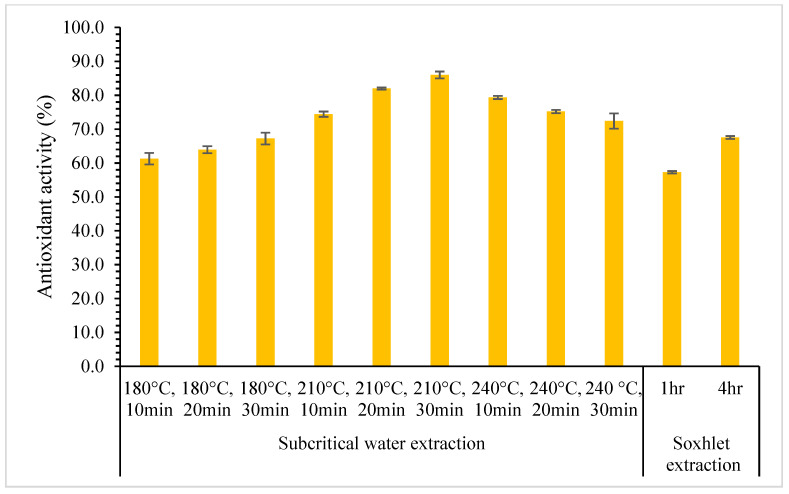
Antioxidant activity comparison between SWE and Soxhlet extraction.

The effectiveness of the subcritical water method to extract phenolic compounds from jackfruit seeds was compared with the Soxhlet extraction. The Soxhlet extraction technique was used as the conventional method with fixed 15% solid loading and was conducted at 1 h and 4 h using pure methanol to extract phenolic compounds. Figure 4 and Figure 5 show the results of total phenolic compounds and antioxidant activity for SWE and Soxhlet extraction. There is a notable difference in the total phenolic compounds and antioxidant activity obtained from the Soxhlet extraction between 1 h (0.53 ± 0.03 mg GAE/100 g) and 4 h (1.20 ± 0.01 mg GAE/100 g) of extraction. The trend is similar to the antioxidant activity extracted using the Soxhlet for 1 h (57.28 ± 0.34%) and 4 h (67.54 ± 0.43%). 

The total phenolic compounds and antioxidant activity results of Soxhlet extraction for 1 h were lower than that using SWE for all process parameters ranging from 180 °C to 240 °C and from 10 to 30 min. For the Soxhlet extraction of 4 h, total phenolic compounds and antioxidant activity results were lower than SWE, with temperatures of 210 and 240 °C. In contrast, results for SWE at 180 °C showed that the total phenolic compounds and antioxidant activity were lower than that obtained from the Soxhlet extraction. This might be due to the dielectric constant of methanol (ε = 31.13) being lower than the dielectric constant of water at 180 °C, in which the dielectric constant of water at 180 °C is approximately ε = 38 [45]. Increasing the extraction temperature above 180 °C may produce a higher value of total phenolic compounds and antioxidant activity since the dielectric constant of water will be lower than methanol. As a result, increasing the temperature of SWE greater than 180 °C makes it feasible to extract phenolic compounds from jackfruit seeds easily at a shorter time compared to Soxhlet extraction.

### 3.4. Surface Morphological Analysis

**Figure 6:**
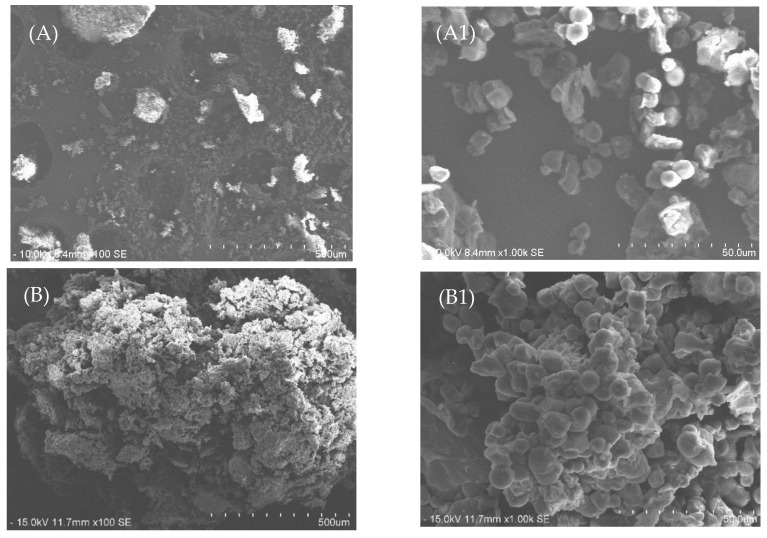

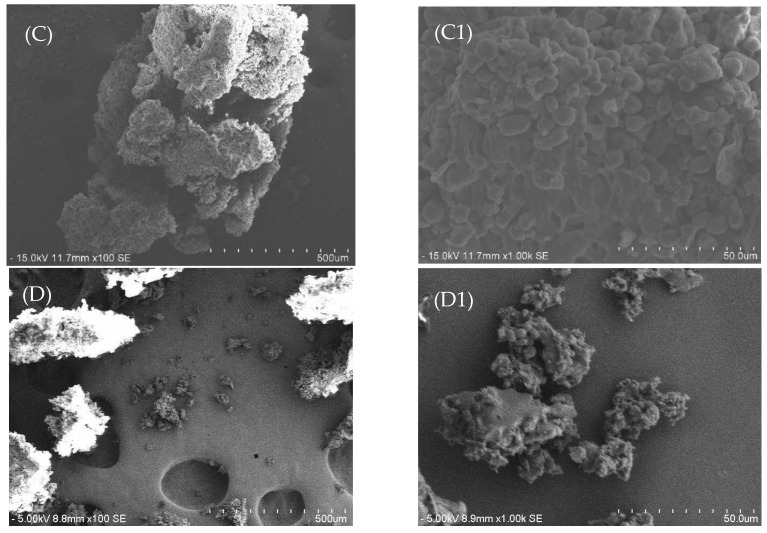
SEM images of jackfruit seed powder (**A**) before extraction, (**B**) residue after 1 h of Soxhlet extraction, (**C**) residue after 4 h of Soxhlet extraction, and (**D**) residue at 210 °C, after 30 min, at 15% solid loading of SWE at ×100 (**A**–**D**) and ×1000 (**A1**–**D1**) magnification.

Scanning electron microscopy (SEM) was used to compare the effects of different extraction methods and conditions on the morphological changes of jackfruit seed powder. In Figure 6(A,A1), the untreated jackfruit seed powder displayed an unbroken and agglomerated structure, resulting in a smoother and spherical-like appearance without visible pores. On the other hand, samples treated with Soxhlet extraction showed swelling but not complete rupture, with most structures remaining intact and adjacent to each other, as depicted in Figure 6(B1,C1). This gentle Soxhlet extraction process, conducted at lower temperatures, resulted in a less physically disrupted and roughened sample surface compared to SWE (subcritical water extraction). These observations support the finding that the total phenolic content and antioxidant activity of jackfruit seeds extracted by conventional Soxhlet extraction are lower when compared to the SWE method.

The surface damage to the jackfruit seed powder treated by SWE was more extensive compared to the untreated sample and samples treated by Soxhlet extraction, resulting in a rougher surface. SWE caused the complete rupture of the sample surface, leading to irregular shapes, and this process is known to segregate and disrupt the cells [36]. In this study, the highest total phenolic compounds were observed at the SWE condition of 210 °C and 30 min, attributed to structural changes caused by high temperatures, resulting in surface roughening. The samples treated by SWE with 15% solid loading (Figure 6(D,D1)) exhibited more detected pores, likely due to the high temperature and pressure during subcritical water extraction, which leads to irregular shape formation and tiny pores. Consequently, the increased pores facilitated higher penetration of phenolic compounds from the jackfruit seed powder. These findings align with previous studies conducted by Zakaria et al. [36] and Rahmah et al. [31], where high temperatures during subcritical water extraction caused cell rupture and the formation of open pores, enabling water to extract phenolic compounds and transfer them through the pores to the water as a solvent phase.

### 3.5. Determination of Functional Group of Jackfruit Seeds Extract

The functional groups of jackfruit seeds extracted from SWE were studied through the wavenumber ranges of Fourier-transform infrared spectroscopy (FTIR) peaks. Phenolic compounds were represented by particular peaks or bands in the FTIR spectrum. 

**Figure 7:**
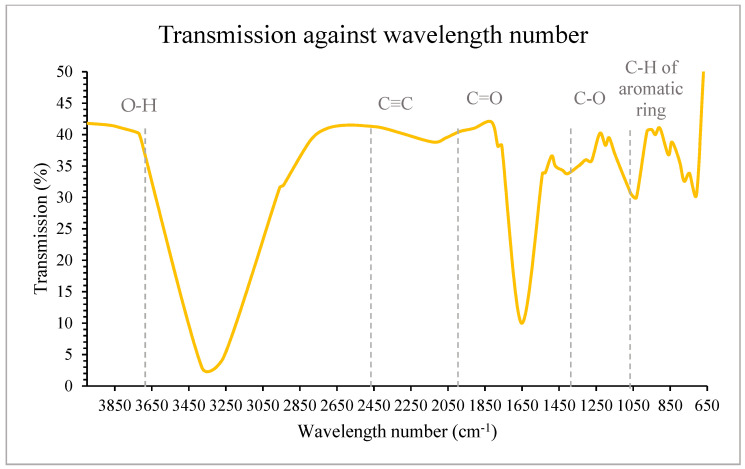
FTIR analysis of subcritical water extract at 210 °C and 30 min.

From Figure 7, a strong and broad absorbance band of jackfruit seeds extracted via SWE at 210 °C, 30 min, and 15% solid loading was identified at a peak of 3380.01 cm^−1^ with significant stretching between 2800 and 3700 cm^−1^. This spectra area relates to the -OH group of carbohydrates, water, and organic acid stretching vibration that is frequently related to carboxylic acid stretching vibrations [46]. This implies that jackfruit seed extract consists of carboxylic acids, exhibits a very strong O-H bond, and indicates the presence of phenolic compounds. A low peak was observed at 2126.73 cm^−1^, which indicates that the C≡C stretching bond of the alkyne is also present. The stretching frequency of mostly alkyne C≡C bonds ranges between 2100 and 2200 cm^−1^.

Another strong absorption peak was observed at 1653.26 cm^−1^, which also corresponds to the -OH group deformation vibrations. There was a slight peak at 1760 cm^−1^ due to the stretching vibrations of functional groups, such as ketone C=O [46]. This suggests that carboxylic acid possesses both the -OH bond and the C=O bond, which means its functional group combines the properties of alcohols and ketones. Two weak bands were observed at 1200 cm^−1^ and 1032 cm^−1^ that belong to the C-O stretching vibration, which responds as the ether functional group, commonly occurring between 1300 cm^−1^ and 1000 cm^−1^ [36].

The spectrum shows that the band that appeared at 707.29 cm^−1^ corresponds to the C-H bond of the aromatic ring between 670 cm^−1^ and 900 cm^−1^. C-H bending vibrations are commonly utilized to identify the degree and kind of substitution. The presence of one or more aromatic rings in a structure is generally easily identified by the vibrations of the C-H and C=C-C rings [31]. Monosubstitution (phenyl) of the aromatic ring group typically has a frequency ranging from 710 cm^−1^ to 690 cm^−1^, due to which it can be presumed that jackfruit seed extract consists of monosubstituted phenyl groups of the aromatic ring. It was suggested that hydroxyl, carboxylic acid, and phenyl functional groups show the presence of phenolic acid in jackfruit seeds. Thus, based on the characterization of phenol (O-H and aromatic structure) and C double bonds, these functional groups also present an antioxidant structure. The identification of functional groups such as OH and carboxylic acid shows that it is the chemical structure of phenolic acids (e.g., gallic acid and ferulic acid), which is also confirmed by HPLC analysis in the next section.

### 3.6. Identification of the Phenolic Acid from Jackfruit Seeds

Phenolic acids describe phenolic compounds as having one carboxylic acid group that occupies phenol moiety and a significant stabilized structure that triggers the H-atom donation, resulting in antioxidant properties via a radical scavenging mechanism [47]. Therefore, it is important to determine the phenolic acids in the jackfruit seed extract that correlate with the antioxidant activity. A previous study by Singh et al. [7] analyzed the phenolic acid contents in different parts of raw and ripe jackfruit using HPLC analysis. They detected gallic acid, ferulic acid, and tannic acid on the ripe fruit seed of jackfruit. Thus, in this study, the phenolic acids of jackfruit seeds were also quantified in the extracted samples using HPLC analysis to identify the main phenolic compounds. However, the data collected (Table 2) from the HPLC analysis detected only two significant peaks corresponding to gallic acid and ferulic acid. The concentrations of gallic acid varied from 0.082 to 1.841 mg/mL, whereas ferulic acid concentrations ranged from 0.041 to 0.068 mg/mL. The highest gallic acid concentration was found at the process parameter of 240 °C at 10 min, while the highest amount of ferulic acid was produced at 180 °C at 10 min. High temperatures and longer extraction times resulted in greater gallic acid concentrations until a certain extraction temperature and time (20 and 30 min at 240 °C). However, further exposure to a high temperature of 240 °C may decrease the concentration of gallic acid. The concentrations of ferulic acid remained relatively stable throughout the process. In contrast to gallic acid, ferulic acid is more susceptible to heat degradation due to its hydroxycinnamic acid classification and the presence of a conjugated double bond structure, making it prone to breakdown at high temperatures. In subcritical conditions (high temperature), the C-glycosidic bond between ferulate ions and glucose could be hydrolyzed to form ferulic acid [48]. Ferulic acid experiences substantial degradation starting at 150 °C and is completely degraded at 250 °C [49]. On the other hand, gallic acid exhibits higher heat resistance compared to ferulic acid. As a tri-hydroxybenzoic acid with a more stable chemical structure, gallic acid can withstand higher temperatures than ferulic acid, although it may still undergo thermal degradation under extreme conditions.

Soxhlet extraction was carried out at 1 h and 4 h. The concentrations of gallic acid of 1 h and 4 h extraction times were 0.138 mg/mL and 0.266 mg/mL, respectively. Ferulic acid concentrations ranged from 0.049 mg/mL (1 h) to 0.046 mg/mL (4 h). When comparing the results between the 1 h and 4 h extraction times, a higher amount of gallic acid was obtained at 4 h, while a higher amount of ferulic acid was found at 1 h. However, the concentration of ferulic acid did not differ significantly between 1 h and 4 h. 

The concentration of gallic acid at the Soxhlet extraction of 4 h (0.266 mg/mL) was higher than the SWE process parameters at 180 °C from 10 min until 30 min (in the range from 0.082 to 0.211 mg/mL). In contrast, when the temperature of SWE increased to 210 °C and 240 °C, the concentration of gallic acid extracted using Soxhlet extraction for 4 h was lower than those in SWE. SWE was more efficient at higher temperatures and shorter exposure times. As discussed in the previous section, this could be due to the water dielectric constant at high temperatures being lower than methanol’s dielectric constant (ε = 31.13). 

On the other hand, the concentrations of ferulic acid obtained by Soxhlet extraction were 0.049 mg/mL at 1 h and 0.046 mg/mL at 4 h. The concentration of ferulic acid varied significantly with different process parameters in subcritical water extraction. This suggests that subcritical water extraction allows for more precise control over extraction conditions, potentially resulting in higher or lower yields, depending on the desired outcome. Soxhlet extraction, being a more traditional method, provided a relatively consistent concentration of ferulic acid regardless of the extraction duration. 

The results of gallic acid and ferulic acid can be related to the total phenolic compounds, which are affected by extraction temperature and time. In comparison to the time-consuming Soxhlet extraction, high temperatures and a shorter SWE treatment time cause the intermolecular hydrogen bonds of water to disintegrate and the dielectric constant of water to drop. In general, jackfruit seeds have a high amount of gallic acid but a low amount of ferulic acid. A similar finding was noted by Singh et al. [7], who also found a higher content of gallic acid (11.3 μg/g) compared to ferulic acid (2.38 μg/g) in raw jackfruit seeds. Overall, these results show that jackfruit seeds have a high concentration of phenolic acids, indicating the nutritional benefits of jackfruit for human health.

## 4. Conclusions

In conclusion, the extraction process temperature and time significantly impacted the total phenolic content and antioxidant activity in jackfruit seeds. Higher temperatures and longer extraction times resulted in increased phenolic content and antioxidant activity, although degradation of some phenolic compounds at high temperatures should be considered. The most favorable results were achieved at 210 °C and 30 min using SWE, yielding total phenolic compounds of 1.84 mg GAE/100 g and antioxidant activity of 86%. SWE’s effectiveness was further evident in surface morphological analysis, where high temperatures caused cell membrane rupture, facilitating phenolic compound extraction. FTIR analysis confirmed the presence of phenolic acids, such as gallic acid and ferulic acid. The highest gallic acid content (1.841 mg/mL) was obtained at 240 °C and 10 min of SWE, while the highest ferulic acid content (0.068 mg/mL) was achieved at 180 °C and 10 min of SWE. In summary, SWE offers an eco-friendly approach to enhance phenolic compound extraction from jackfruit seeds, with water serving as a feasible alternative to organic solvents, such as methanol. SWE’s efficiency was evident, with the highest total phenolic compounds and antioxidant activity achieved in just half an hour compared to Soxhlet extraction.

## Figures and Tables

**Table 1:** Total phenolic content and antioxidant activity of jackfruit seed extract using SWE at 15% solid loading.

Run Order	Temperature	Time	Total Phenolic Content	Antioxidant Activity
	°C	min	mg GAE/100 g	%
1	180	10	0.84 ± 0.04 ^e^	61.29 ± 1.72 ^f^
2	180	20	1.07 ± 0.00 ^d^	63.96 ± 1.03 ^e,f^
3	180	30	1.17 ± 0.07 ^d^	67.23 ± 1.72 ^e^
4	210	10	1.34 ± 0.01 ^c^	74.421 ± 0.78 ^c,d^
5	210	20	1.58 ± 0.01 ^b^	82.021 ± 0.32 ^a,b^
6	210	30	1.84 ± 0.02 ^a^	86.00 ± 1.01 ^a^
7	240	10	1.58 ± 0.00 ^b^	79.36 ± 0.47 ^b,c^
8	240	20	1.41 ± 0.02 ^c^	75.20 ± 0.49 ^c,d^
9	240	30	1.41 ± 0.04 ^c^	72.39 ± 2.25 ^d^

Note. Each group is allocated a letter (a, b, c, d, e and f), which indicates that groups with the same letter are not significantly different.

**Table 2:** Concentration of phenolic acid using SWE and Soxhlet extraction.

Extraction Methods	Process Parameters	Concentration of Gallic Acid	Concentration of Ferulic Acid
		(mg/mL)	(mg/mL)
SWE 15% solid loading	180 °C, 10 min	0.082 ± 0.020	0.068 ± 0.027
	180 °C, 20 min	0.154 ± 0.070	0.042 ± 0.001
	180 °C, 30 min	0.211 ± 0.112	0.042 ± 0.000
	210 °C, 10 min	0.418 ± 0.175	0.041 ± 0.000
	210 °C, 20 min	1.266 ± 0.292	0.043 ± 0.000
	210 °C, 30 min	1.176 ± 0.335	0.045 ± 0.003
	240 °C, 10 min	1.841 ± 0.082	0.042 ± 0.000
	240 °C, 20 min	0.664 ± 0.003	0.046 ± 0.002
	240 °C, 30 min	0.809 ± 0.037	0.054 ± 0.002
Soxhlet extraction	1 h	0.138 ± 0.029	0.049 ± 0.003
	4 h	0.266 ± 0.094	0.046 ± 0.004

## Data Availability

The datasets generated for this study are available on request to the corresponding author.
